# Risk and Determinants of Dementia in Patients with Mild Cognitive Impairment and Brain Subcortical Vascular Changes: A Study of Clinical, Neuroimaging, and Biological Markers—The VMCI-Tuscany Study: Rationale, Design, and Methodology

**DOI:** 10.1155/2012/608013

**Published:** 2012-04-08

**Authors:** Anna Poggesi, Emilia Salvadori, Leonardo Pantoni, Giovanni Pracucci, Francesca Cesari, Alberto Chiti, Laura Ciolli, Mirco Cosottini, Alessandra Del Bene, Nicola De Stefano, Stefano Diciotti, Maria Teresa Dotti, Andrea Ginestroni, Betti Giusti, Anna Maria Gori, Serena Nannucci, Giovanni Orlandi, Francesca Pescini, Raffaella Valenti, Rosanna Abbate, Antonio Federico, Mario Mascalchi, Luigi Murri, Domenico Inzitari

**Affiliations:** ^1^Department of Neurological and Psychiatric Sciences, University of Florence, Largo Brambilla 3, 50134 Florence, Italy; ^2^Department of Medical and Surgical Critical Care, University of Florence, 50134 Florence, Italy; ^3^Department of Neurosciences, University of Pisa, 56126 Pisa, Italy; ^4^Department of Neurological and Behavioral Sciences, University of Siena, 53100 Siena, Italy; ^5^Radiodiagnostic Section, Department of Clinical Physiopathology, University of Florence, 50134 Florence, Italy

## Abstract

Dementia is one of the most disabling conditions. Alzheimer's disease and vascular dementia (VaD) are the most frequent causes. Subcortical VaD is consequent to deep-brain small vessel disease (SVD) and is the most frequent form of VaD. Its pathological hallmarks are ischemic white matter changes and lacunar infarcts. Degenerative and vascular changes often coexist, but mechanisms of interaction are incompletely understood. The term mild cognitive impairment defines a transitional state between normal ageing and dementia. Pre-dementia stages of VaD are also acknowledged (vascular mild cognitive impairment, VMCI). Progression relates mostly to the subcortical VaD type, but determinants of such transition are unknown. Variability of phenotypic expression is not fully explained by severity grade of lesions, as depicted by conventional MRI that is not sensitive to microstructural and metabolic alterations. Advanced neuroimaging techniques seem able to achieve this. Beside hypoperfusion, blood-brain-barrier dysfunction has been also demonstrated in subcortical VaD. The aim of the Vascular Mild Cognitive Impairment Tuscany Study is to expand knowledge about determinants of transition from mild cognitive impairment to dementia in patients with cerebral SVD. This paper summarizes the main aims and methodological aspects of this multicenter, ongoing, observational study enrolling patients affected by VMCI with SVD.

## 1. Introduction

Dementia is one of the most disabling conditions affecting older people. The most frequent cause is Alzheimer's disease (AD) which is attributed to brain cortex degeneration. In Italy one-third of dementia cases are due to vascular dementia (VaD), a term that encompasses a few subtypes, among which subcortical VaD consequent to deep brain small vessel disease (SVD) is the most frequent [1]. Its pathological hallmarks are ischemic white matter changes, caused by hypoperfusion and lacunar infarcts [2]. These changes can progress over time silently, a profile similar to that occurring in AD. In the old brain degenerative and vascular changes may often coexist and possibly interact. However, the exact mechanisms of such interaction are still incompletely understood [3]. Data suggest that vascular factors play a role also in AD [4]. How they contribute to both dementia types is a relevant issue, as they can be treated, favorably altering the progression of cognitive decline. In the neurovascular unit, vessels and cells, including neurons, closely interact, pointing to endothelial dysfunction as a key element for explaining neuronal damage associated with both subcortical VaD and AD.

The term mild cognitive impairment (MCI) defines a transitional state between normal ageing and dementia and is thought to anticipate dementia [5]. Subjects with MCI, compared to those without, progress to AD at a rate ten times greater [6]. Pre-dementia stages of VaD are also acknowledged: this condition is referred to as vascular mild cognitive impairment (VMCI) [7]. In the Canadian Health and Aging Study 46% of patients with VMCI developed dementia after 5 years [8]. Progression relates mostly to the subcortical VaD type. The determinants of this transition are not fully elucidated: it is not clear whether it is mainly driven by vascular, degenerative processes, or by their interaction. The increase in white matter changes and the accumulation of small infarcts proved to be associated with the cognitive decline [7]. Data show that brain atrophy is also associated with cognitive deterioration [9].

Phenotypic expression of subcortical VaD is variable. Variability is not fully explained by severity grade of lesions, as depicted by conventional MRI. This seems not enough sensitive in differentiating axonal loss and glia damage from intact structures. Furthermore, it does not provide pathophysiological details about changes ongoing in both parenchyma and vessels. Advanced techniques such as magnetization transfer, diffusion, and spectroscopy seem able to yield more in-depth information about the extent of tissue damage, its microstructural nature, and the underlying metabolic alterations [10]. Beside hypoperfusion, blood brain-barrier dysfunction has been also demonstrated in subcortical VaD [11, 12]. Since many factors associated with subcortical VaD are treatable, it seems of the utmost importance to expand the knowledge about determinants and mechanisms underlying the transition from VMCI to dementia. Estimating the relative weight of the predictive effect for each significant determinant (among the clinical, functional, imaging, and biological potential markers) of the transition may allow to evaluate the risk of dementia and to describe the risk-factor profile in individual patients with subcortical MCI, thus favoring tailored and appropriate preventive strategies.

During the last 10 years many clinical and functional outcomes in elderly patients with subcortical vascular changes have been investigated through a European multicenter collaboration called LADIS (leukoaraiosis and disability) Study [13]. The LADIS study, supported by the European Union, has been carried out in 11 European high-quality centers, coordinated by the Department of Neurological and Psychiatric Sciences of the University of Florence. Concerning the main study outcome, that is, transition to disability, results have shown that the risk of transition or death was more than twofold higher in patients with severe white matter changes compared to those with mild degrees [14]. Some vascular risk factors also predicted the transition. A review summarizing the LADIS main results has recently been published [15]. White matter changes also had an effect on cognitive decline and dementia. Cross-sectional baseline data analysis indicated that white matter changes and medial temporal lobe atrophy were independently associated with cognitive impairment [9]. At multivariable analysis, risk factors and the conventional MRI features were able to explain only part of the prediction of either disability or dementia [16]. It was thus decided to perform a new study, the VMCI-Tuscany Study, with the main aims of: (1) estimating the net and multivariable effect in predicting the transition from VMCI to dementia studying a large set of both conventional and nonconventional clinical, neuro-imaging, and biological markers of SVD; (2) assessing the vascular and degenerative components in the determination of such transition, that is, their relative contribution and their possible interaction; (3) generating from the results of the multivariable predictive model a diagnostic algorithm able to determine the risk of transition in individual patients with VMCI with SVD.

## 2. Methods

The VMCI-Tuscany Study foresees the collaboration of the three university hospital centers in Tuscany: Florence, Pisa, and Siena (Department of Neurological and Psychiatric Sciences, University of Florence, Unit of Neurology and Neurophysiopathology, Azienda Ospedaliero-Universitaria Pisana, and Unit of Neurology and Neurometabolic Diseases, Azienda Universitaria Ospedaliera Senese) where the enrolment and assessment of patients with VMCI and SVD is actually ongoing. In order to reach the prespecified number of patients in the foreseen time period, the project has been promoted at all the regional, both neurological and geriatric, hospital units (Figure 2).

### 2.1. Inclusion Criteria

**Figure 1:**
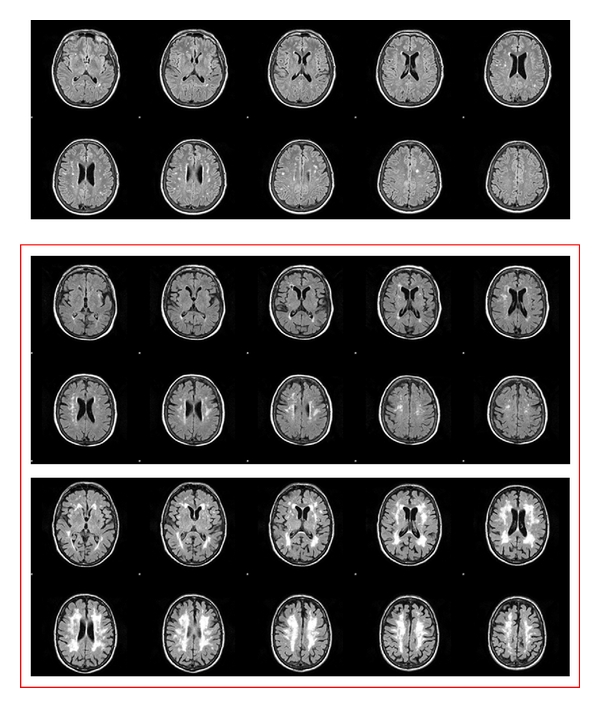
Cerebral white matter changes: examples of mild, moderate, and severe groups according to the modified Fazekas' scale. Only patients with moderate or severe degrees were included in the study.

**Figure 2:**
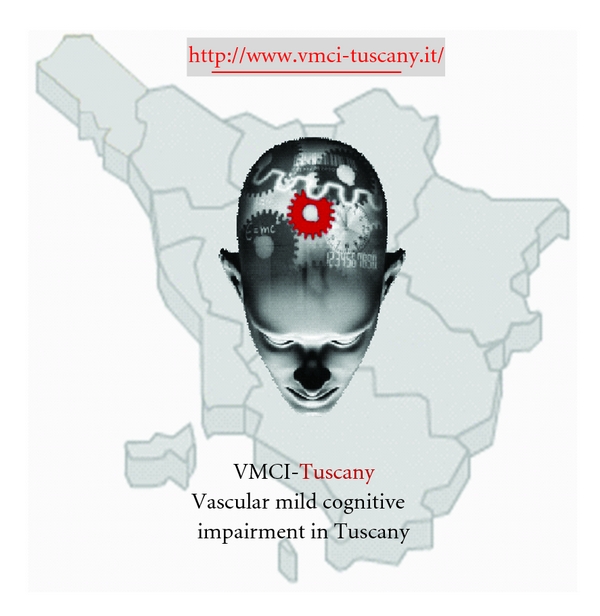
List of participating centers and personnel in the VMCI-Tuscany. *University of Florence*: (Coordinating Center): Domenico Inzitari (study coordinator), Rosanna Abbate, Manuela Bandinelli, Maria Boddi, Francesca Cesari, Laura Ciolli, Mirella Coppo, Alessandra Del Bene, Stefano Diciotti, Andrea Ginestroni, Betti Giusti, Anna Maria Gori, Mario Mascalchi, Serena Nannucci, Leonardo Pantoni, Marco Pasi, Francesca Pescini, Anna Poggesi, Giovanni Pracucci, Emilia Salvadori, Raffaella Valenti. *University of Pisa*: Luigi Murri, Ubaldo Bonucelli, Paolo Cecchi, Alberto Chiti, Mirco Cosottini, Giovanni Orlandi, Cristina Pagni, Gabriele Siciliano, Gloria Tognoni. *University of Siena*: Antonio Federico, Nicola De Stefano, Maria Teresa Dotti, Patrizia Formichi, Claudia Gambetti, Antonio Giorgio, Francesca Rossi, Laura Stromillo, Enza Zicari. *Tuscany region: Arezzo* (Paolo Zolo, Alessandro Tiezzi); *Empoli* (Elisabetta Bertini, Stefania Brotini, Leonello Guidi, Maria Lombardi, Stefania Mugnai, Antonella Notarelli); *Florence* (Laura Bracco, Massimo Cadelo, Renzo Cisbani, Luciano Gabbani, Guido Gori, Lorella Lambertucci, Luca Massacesi, Enrico Mossello, Marco Paganini, Maristella Piccininni, Francesco Pinto, Claudia Pozzi, Sandro Sorbi, Gaetano Zaccara); *Grosseto* (Tiziano Borgogni, Mario Mancuso, Roberto Marconi); *Lucca* (Monica Mazzoni, Marco Vista); *Livorno* (Giuseppe Meucci, Giovanna Bellini); *Massa Carrara* (Luciano Gabrielli); *Pisa* (Cristina Frittelli, Renato Galli, Gianna Gambaccini); *Pistoia* (Stefano Bartolini, Carlo Biagini, Veronica Caleri, Paola Vanni); *Prato* (Donatella Calvani, Carla Giorgi, Stefano Magnolfi, Pasquale Palumbo, Carlo Valente); *Siena* (Alessandro Rossi, Rossana Tassi, Stefania Boschi); *Viareggio* (Filippo Baldacci; Ubaldo Bonuccelli).

To be included, patients have to be classified as affected by VMCI with SVD according to the following criteria: (1) MCI defined according to Winblad et al. criteria [17], and (2) evidence on MRI of subcortical vascular lesions of moderate to severe degrees of white matter changes (WMC), according to the modified version of the Fazekas scale [13] (Figure 1). Each patient gives a written informed consent, and the study has been approved by local ethical committees.

### 2.2. Exclusion Criteria

Exclusion criteria are inability or refusal to undergo cerebral MRI, and inability to give an informed consent.

Sample size was estimated based on data about transition to dementia obtained in the LADIS Study. In the LADIS sample, patients with baseline mild cognitive impairment and moderate to severe WMC, had an annual rate of transition to dementia of 10%. Three hundred patients were considered the minimal number necessary to carry out multivariate analyses of potential predictors of transition to dementia.

#### 2.2.1. Baseline Assessment

According to the study protocol, each patient enrolled undergoes an extensive clinical assessment and an MRI examination and permits the collection of blood samples for the determination of plasma biomarkers. Tables 1, 2, and 3 summarize main objectives and instruments for the clinical, neuroimaging, and laboratory assessments, respectively.

### 2.3. Clinical Assessment (Table 1)

Social background and medical history are collected according to a structured and comprehensive questionnaire administered to the patient by trained personnel. This includes information on demographic characteristics, education (expressed as years of schooling), occupational status, longest job in life, marital status, living conditions, lifestyle habits (alcohol consumption, smoking, physical activity), and vascular risk factors. Furthermore, patients are specifically asked if they experience gait, memory, visual, or hearing problems, or bladder disturbances. Several other age-related comorbidities are assessed with the aim of controlling for their effect on the main study outcome. All possible medical information and records were collected and reviewed during the interview in order to reach or confirm the diagnosis of the diseases under investigation. If needed, contact with the family doctor can also be undertaken. All conditions were defined according to the currently most widely accepted criteria, selected after a systematic literature search. The whole set of criteria is reported in a specifically developed manual and distributed to all participating centers. Information is also collected about thyroid diseases, head injuries, falls in the last year. Data on family history of migraine, epilepsy, psychiatric disturbances, stroke, dementia, venous thrombosis, diabetes mellitus, hypertension, and hyperlipidemias are also recorded. Currently used drugs are registered in a structured way.

Blood pressure, weight, and height measurements are recorded, and a standard neurological examination is performed. The absence or presence of the following neurological examination abnormalities is assessed: upper motor neuron signs, extrapyramidal signs, cerebellar signs, cortical signs (language deficits and/or visual field abnormalities), pseudobulbar signs (hypophonia and/or dysphagia and/or dysarthria), and primitive reflexes (one or more of the following: grasp reflex, palmomental reflex, forced laughing and crying, snout reflex, glabellar tapping). Speed of finger taps (patient taps thumb with index finger in rapid succession with widest amplitude possible) is assessed for each hand separately and scored as normal, mild slowing, moderately impaired, severely impaired, or barely able to perform the task (range 1 to 5). Gait and stance are coded as normal or abnormal. Concerning motor function, a standardized assessment is performed by means of the short physical performance battery (SPPB), and two additional simple measures of gait and balance, that is, gait velocity and single leg stance time, as already used in the LADIS study [18–20, 34].

Each patient is assessed by means of an extensive neuropsychological evaluation according to the specifically developed test battery. How the neuropsychological test battery was developed and the way it allows automation and standardization of the scoring procedures will be reported in a separate paper. In brief, the cognitive functions assessed include global mental functioning, orientation, memory, attention, executive functions, language, speed and motor control. The tests used are reported in Table 1 [21–30]. The geriatric depression scale (GDS) is used for the assessment of mood [31]. Functional status is measured by means of the activities of daily living scale and the instrumental activities of daily living scale [32, 33]: these 2 scales are addressed to the patient's caregiver, given the longitudinal design of the study and the possible transition to dementia of the patient.

 In order to increase consistency and homogeneity in the assessments, all investigators participated to at least one training session and were provided with a specifically developed handbook with guidelines for the correct use of criteria and tools.

### 2.4. MRI Examination (Table 2)

Patients are examined on clinical 1.5 T system (Intera, Philips Medical System, Best, The Netherlands) with 33 mT/m gradients capability and a head coil with SENSE technology. After scout, the examination protocol includes a sagittal T1 weighted 3D T1-weighted turbo gradient echo sequence (repetition time (TR) = 8.1 ms, echo time (TE) = 3.7 ms, flip angle = 8°, inversion time (TI) = 764 ms, field of view (FOV) = 256 mm, matrix size = 256 × 256, 160 contiguous slices, slice thickness = 1 mm) axial FLAIR sequence (TR = 11000 ms, TE = 140 ms, inversion time (TI) = 2800 ms, FOV = 230 mm, matrix size = 320 × 216, contiguous slices, slice thickness = 5 mm), and axial single-shot echo planar imaging sequence (TR = 9394 ms, TE = 89 ms, FOV = 256 mm, matrix size = 128 × 128, 50 slices, slice thickness = 3 mm, no gap, NEX = 3; diffusion sensitizing gradients applied along 15 non-collinear directions using b value of 0 (b0 image) and 1000 s/mm^2^) for DTI. A T2* weighted echo planar imaging sequence is used for the functional MRI. Details of such experiments are reported elsewhere.

### 2.5. Laboratory Analyses (Table 3)

Tests for plasma biomarkers will be performed centrally, in Florence, Center for Thrombosis, after the collection of blood samples of all recruited patients will be completed. Laboratory investigations will then include the determination of those plasma biomarkers which, according to literature review, have been found to be associated with white matter changes. At the moment, the possible set of plasma biomarkers is composed of markers of endothelial function, polymorphisms associated with cerebral small vessels disease, and pro- and anti-inflammatory molecules (see Table 3 for details).

#### 2.5.1. Follow-Up Assessment

The longitudinal design of the VMCI-Tuscany study encompass a clinical, functional, and neuropsychological assessment, according to the main study protocol already used at baseline, performed yearly after enrollment, for two years consecutively. The MRI assessment is performed at baseline and then repeated after two years, at the end of the study. This second MRI is planned with the aim of evaluating possible progression of both vascular and nonvascular lesions and of studying this progression in correlation with the evolution of clinical/functional deficits and the onset of dementia.

### 2.6. Outcome Measures

The main study outcomes will be the diagnosis of dementia and the occurrence of cognitive decline. Dementia diagnosis will be made according to DSM-IV criteria [35]. The diagnosis of vascular dementia will be made according to specific criteria [36]; the diagnosis of the main subtypes of vascular dementia will be performed for consensus according to clinical and neuroimaging characteristics. AD diagnosis will be performed according to the clinical research criteria [37]. Staging of dementia severity will be performed according to clinical dementia rating scale [38]. Cognitive decline will be evaluated as a decrease in the compound scores for each cognitive domain.

### 2.7. Data Management

Data collected in each center is entered into an electronic database on a specifically developed website (http://www.vmci-tuscany.it/). This allows the systematic and rapid control of completeness and online quality of data in real time.

## 3. Results

The study began on October 15, 2010. The first four months of the study have been dedicated to the preparation of all the study instruments and training of the participating personnel. During this first phase, the definitive project protocol has been established and shared by all the investigators, and the homogeneity of the clinical-functional and MRI sequences parameters have been tested across the three centres and investigators. The dedicated website for communications, storage of study instruments, and database for data collection has been created and is operative (http://www.vmci-tuscany.it/).

On February 15, 2011, the enrollment of patients was started. Up to now, eight months after the beginning of the study, 76 patients with moderate to severe WMC have been evaluated according to the entire study protocol. Mean age is 75.2 ± 7.4, 47 (62%) are males, and mean education, expressed as years of schooling, is 7.5 ± 3.9. Mean MMSE score is 26.6 ± 2.7, and mean MoCA score is 19.6 ± 5.0.

The end of the enrollment is scheduled for the end of 2012.

## 4. Discussion

The VMCI-Tuscany Study is one of the first longitudinal studies assessing determinants of the transition from a stage of mild cognitive impairment to dementia. For the first time the study will focus on a particular form of cerebrovascular disease as a cause of cognitive decline, that is, cerebral small vessel disease. Up to now, the study protocol showed a good feasibility and applicability in patients with VMCI and SVD. The first aim of the study is to investigate a large set clinical, neuroimaging, and biological markers of cerebral small vessel disease in predicting the transition from a stage of mild cognitive impairment to dementia. Secondly, the study will permit the assessment of the relative contribution, and possible interaction, of vascular and degenerative components in the determination of such transition. Last, but not least, final results of the multivariable predictive model will enable the generation a diagnostic algorithm able to determine the risk of transition to dementia in individual patients with VMCI and SVD.

Data from the literature show that the clinical and functional status in subjects with the same grade of WMC may be variable, suggesting the presence of “hidden” factors in determining the clinical picture [39]. Despite the known role of SVD in the determination of cognitive impairment, its net weight remains to be appreciated. For example it is known that some patients with similar visible SVD burden have different clinical outcomes, hinting at the presence of additional, uncovered aspects that may contribute to this transition. This uncovered aspect may relate to the same pathogenic process (SVD) but be undetected under routine assessment (e.g., conventional MRI) and instead be detected by new techniques [39]. The morphological alterations identified on conventional neuroimaging do not appear sufficient to explain the clinical-functional phenotype which is frequently variable for typology and severity also in presence of overlapping neuroimaging findings. A number of studies have investigated the association between white matter lesions extension, evaluated by means of visual scales or volume measurements, and functional, cognitive or motor disturbances, sometimes with inconsistent results. A limitation of these studies is that the signal alterations on conventional neuroimaging may be due to different degrees of parenchymal damage, from more or less severe axonal loss to a glia damage with intact nervous fibers. Conventional morphological neuroimaging is not able to provide details about pathophysiological processes within the cerebral parenchyma and vessels. Preliminary studies using advanced neuroimaging techniques such as magnetization transfer, diffusion, and spectroscopy have suggested the possibility to acquire more in-depth information about the microstructural tissue damage and underlying metabolic alterations and the association with the cognitive performances evaluating both white matter lesions revealed by conventional neuroimaging and normal-appearing white matter. However, such data have been obtained from small cross-sectional studies and without considering the influence of other frequently coexisting lesions such as lacunar infarcts and cortical atrophy.

Knowledge about determinants of the transition to dementia in patients with VMCI and SVD is essential to the identification of potential preventive and therapeutic targets, contributing to reduce the burden (on both the ethical and economical grounds) of disability in the elderly. This represents one of the greatest challenges for social and health systems in our ageing society. Moreover, results from the project may provide both health systems and professionals with a validated instrument (i.e., a diagnostic algorithm generated from the multivariable prediction models) for estimating the risk of transition to dementia in individual patients tailoring the appropriate preventive and therapeutic strategy. Finally, the multivariable prediction of the risk of cognitive decline and dementia in this population may represent an essential methodological background for designing and sample-sizing-controlled clinical trials in the selective clinical setting.

## Figures and Tables

**Table 1:** Instruments for patients' assessment in the VMCI-Tuscany study protocol.

	Instruments/methods	References
Clinical assessment	Social background and medical history according to structured protocol	—
	Standard cardiovascular and neurological examination according to structured protocol	—

Motor performances	Short physical performance battery (SPPB)	[18]
	Single leg stance	[19]
	Gait velocity	[20]

Cognitive performances	Mini mental state examination (MMSE)	[21]
	Montreal cognitive assessment battery (MoCA)	[22]
	Rey auditory-verbal learning test	[23]
	Trail making test (A and B)	[24]
	Color word stroop test	[25]
	Symbol digit modalities test	[26]
	Visual search	[27]
	Phonemic and semantic fluency	[28]
	Rey-Osterrieth complex figure	[29]
	Short story recall test	[30]

Mood	Geriatric depression scale (GDS-15)	[31]

Global functioning	Activities of daily living (ADL) scale Instrumental activities of daily living (IADL) scale	[32] [33]

**Table 2:** MRI protocol in the VMCI-Tuscany Study.

Sequences	
(i) Axial high-resolution contiguous 3D T1-weighted images with isotropic voxels (MPRAGE sequence)	
(ii) Axial T2-weighted images (FLAIR sequence)	
(iii) Diffusion tensor imaging (DTI)	
(iv) Functional MRI (motor or cognitive tasks)	
(v) T2*-sensitive echo-planar imaging (EPI) sequence	

**Table 3:** Plasma biomarkers under investigation in the VMCI-Tuscany Study.

*Markers of endothelial function*	von Willebrand factor, tissue factor, tissue factor pathway inhibitor, intercellular adhesion molecule-1 and count of the number of circulating endothelial progenitor cells by cytofluorimetric method using monoclonal antibodies anti-staminal cells markers (CD34 and AC133) and anti-endothelial cell markers (VEGFR2)
*Polymorphisms associated with cerebral small vessels disease*	Genes coding metalloproteases and their inhibitors, renin-angiotensin system components, genes coding endothelial nitroxide synthase

*Pro- and anti-inflammatory molecules*	interleukin-6, interleukin-1-RA, interleukin-8, interleukin-10, interleukin-18, and C-reactive protein
